# *Usp9x*-deficiency disrupts the morphological development of the postnatal hippocampal dentate gyrus

**DOI:** 10.1038/srep25783

**Published:** 2016-05-16

**Authors:** Sabrina Oishi, Susitha Premarathne, Tracey J. Harvey, Swati Iyer, Chantelle Dixon, Suzanne Alexander, Thomas H. J. Burne, Stephen A. Wood, Michael Piper

**Affiliations:** 1 The School of Biomedical Sciences, The University of Queensland, Brisbane, QLD, 4072, Australia; 2 The Eskitis Institute for Drug Discovery, Griffith University, Brisbane, QLD, 4111, Australia; 3 Queensland Centre for Mental Health Research, The Park Centre for Mental Health, Richlands, QLD, 4077, Australia; 4 Queensland Brain Institute, The University of Queensland, Brisbane, QLD, 4072, Australia

## Abstract

Within the adult mammalian brain, neurogenesis persists within two main discrete locations, the subventricular zone lining the lateral ventricles, and the hippocampal dentate gyrus. Neurogenesis within the adult dentate gyrus contributes to learning and memory, and deficiencies in neurogenesis have been linked to cognitive decline. Neural stem cells within the adult dentate gyrus reside within the subgranular zone (SGZ), and proteins intrinsic to stem cells, and factors within the niche microenvironment, are critical determinants for development and maintenance of this structure. Our understanding of the repertoire of these factors, however, remains limited. The deubiquitylating enzyme USP9X has recently emerged as a mediator of neural stem cell identity. Furthermore, mice lacking *Usp9x* exhibit a striking reduction in the overall size of the adult dentate gyrus. Here we reveal that the development of the postnatal SGZ is abnormal in mice lacking *Usp9x*. *Usp9x* conditional knockout mice exhibit a smaller hippocampus and shortened dentate gyrus blades from as early as P7. Moreover, the analysis of cellular populations within the dentate gyrus revealed reduced stem cell, neuroblast and neuronal numbers and abnormal neuroblast morphology. Collectively, these findings highlight the critical role played by USP9X in the normal morphological development of the postnatal dentate gyrus.

Neurogenesis encapsulates the tightly controlled process by which neural stem cells give rise to neurons within the nervous system. Neural stem cells preserve the capacity for self-renewal while also undergoing proliferation and differentiation into lineage-specific post-mitotic cells^1^. Although the vast majority of neural stem cells are depleted soon after birth, two main locations within the cerebral cortex retain a population of these cells that support continued neurogenesis throughout life^2^,^3^. These regions, called ‘neurogenic niches’, are the subventricular zone lining the lateral ventricles^4^ and the subgranular zone (SGZ) of the hippocampal dentate gyrus^5^. A highly dynamic network of intrinsic and extrinsic factors act in concert to maintain the balance between quiescence of neural stem cells and their proliferation and subsequent neurogenic differentiation within the adult brain^1^. Disruption to these processes can result in neurodevelopmental diseases, as well as contributing to neurodegenerative disorders in adulthood. Therefore, it is critical to understand the mechanisms underpinning maintenance of the neurogenic niches within the postnatal and adult brain.

The post-translational modification of proteins plays a critical role in cellular and tissue homeostasis. The reversible nature of this process enables cells to rapidly adapt and modify the activity of signaling pathways in response to extrinsic and intrinsic stimuli. For instance, protein ubiquitylation and deubiquitylation plays a key role in mediating protein stability, trafficking and degradation^6^. Whilst ubiquitylation of proteins is well known to regulate protein stability, deubiquitylating proteins (DUBs) are also critical components of cellular homeostasis, and are known to contribute to many developmental paradigms^6^. The site-specific DUB ubiquitin-specific protease 9X (USP9X) is a prime example of this, as it has been shown to contribute to processes including protein trafficking and endocytosis^7^,^8^, cellular polarity^9^,^10^ and cell death^11^,^12^. USP9X has also been implicated in maintaining embryonic, haematopoetic and neural stem cell identity^13^,^14^. With respect to the nervous system, USP9X is expressed by neural stem cells in the developing cerebral cortex, as well as within the adult neurogenic niches^15^, and it promotes neural stem cell self-renewal *in vitro*^9^. *In vivo,* the conditional ablation of *Usp9x* from neural stem cells culminates in deficits in cortical architecture and abnormalities within the germinal ventricular zone of the developing cerebral cortex^15^. The most prominent phenotype of *Usp9x*-deficient mice is, however, the dramatic reduction in the size of the adult hippocampus^15^. The underlying cellular phenotypes contributing to this reduction in hippocampal size remain undefined.

Interestingly, the hippocampal phenotype of mice lacking *Usp9x* just prior to birth is subtle^9^, suggesting that postnatal hippocampal development is abnormal in these mice. The first three weeks after birth in rodents are critical for hippocampal morphogenesis, as this is the time during which the SGZ of the dentate gyrus resolves and matures^16^. Here we analysed postnatal and adult mice in which *Usp9x* had been removed specifically from neural progenitor cells within the dorsal telencephalon (via conditional ablation using an *Emx1*-*Cre* allele) to study the trajectory of postnatal hippocampal development in mice lacking this DUB. We reveal that hippocampal size, as well as the length of the blades of the dentate gyrus, is significantly reduced in comparison to control littermate mice from as early as postnatal day (P) 7. Furthermore, there were significantly fewer quiescent neural progenitor cells within *Usp9x*-deficent mice, as well as a dramatic reduction in the number of neuroblasts expressing the microtubule-associated protein doublecortin (DCX). Morphologically, those neuroblasts that were produced in mutant mice exhibited severe deficits in the extension and elaboration of dendritic processes. Collectively, these findings illustrate that abnormal production and/or maintenance of the neural progenitor cell population, coupled with reduced neuroblast numbers, underlie, at least in part, the reduced dentate gyrus size in mice lacking *Usp9x*, findings that are consistent with this DUB acting to maintain stem cell identity^9^,^13^,^14^.

## Results

**Figure 1:**
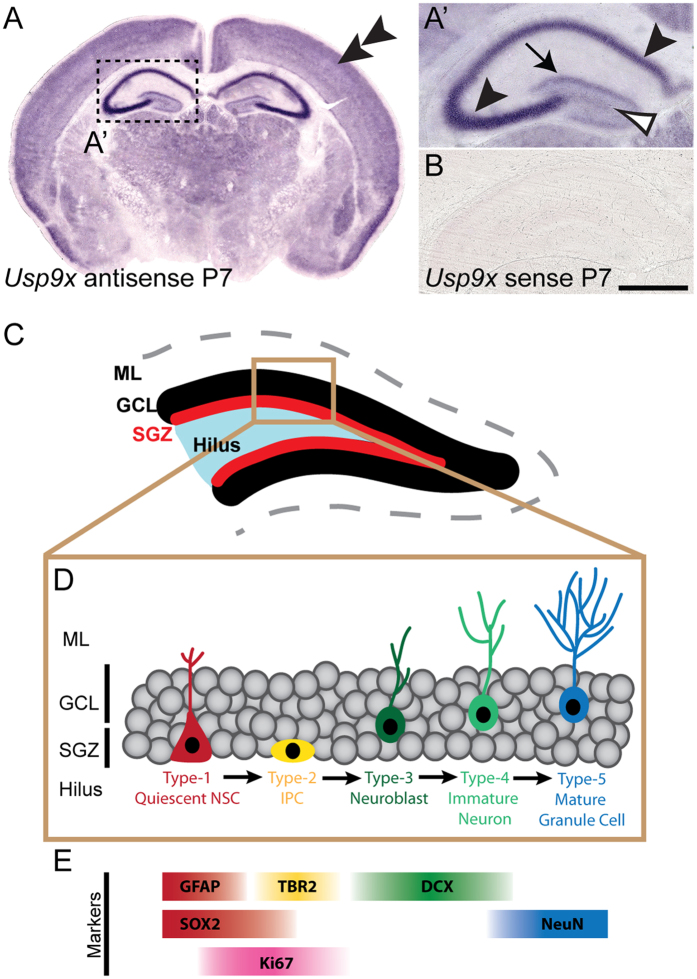
*Usp9x* is expressed within the postnatal hippocampus. (**A**) Coronal section of a P7 mouse brain at the level of the hippocampus, showing the expression of *Usp9x. Usp9x* mRNA is expressed within the cortical plate (double arrowhead). A higher magnification view of the boxed region is shown in A’, revealing that *Usp9x* is expressed within the CA regions (arrowheads), blades of the dentate gyrus (arrow) and the hilus (open arrowhead) of the hippocampus. (**B**) *In situ* hybridisation with a sense *Usp9x* probe did not reveal any specific staining. (**C**) Schematic showing the anatomical organisation of the postnatal and adult dentate gyrus, which includes the molecular layer (ML), the granule cell layer (GCL), the subgranular zone (SGZ) and the hilus. Neural stem cells (NSCs) are found within the SGZ. (**D**) Neurogenesis in the dentate gyrus begins with quiescent NSCs (Type-1 cells) entering the cell cycle and proliferating, producing intermediate progenitor cells (IPCs; Type-2 cells) and then neuroblasts (Type-3 cells). These neuroblasts develop into immature neurons (Type-4 cells), which ultimately give rise to mature granule cells (Type-5 cells). (**E**) Different cellular populations within the dentate gyrus can be identified via the expression of proteins including GFAP, SOX2, Ki67, TBR2, DCX and NeuN. Scale bar in (**B**): A −800 μm; A’, B −150 μm.

USP9X expression has previously been reported within the dentate gyrus and within the cornu ammonis (CA) regions of the adult mouse hippocampus^9^,^17^. Postnatally, we revealed a similar pattern of *Usp9x* expression. At P7 *Usp9x* mRNA was expressed strongly within the stratum pyramidale of the CA3 subfield, and was also observed within this area of the CA1 subfield, as well as within the hilus and blades of the dentate gyrus (Fig. 1). A sense riboprobe did not reveal any specific staining (Fig. 1).

**Figure 2:**
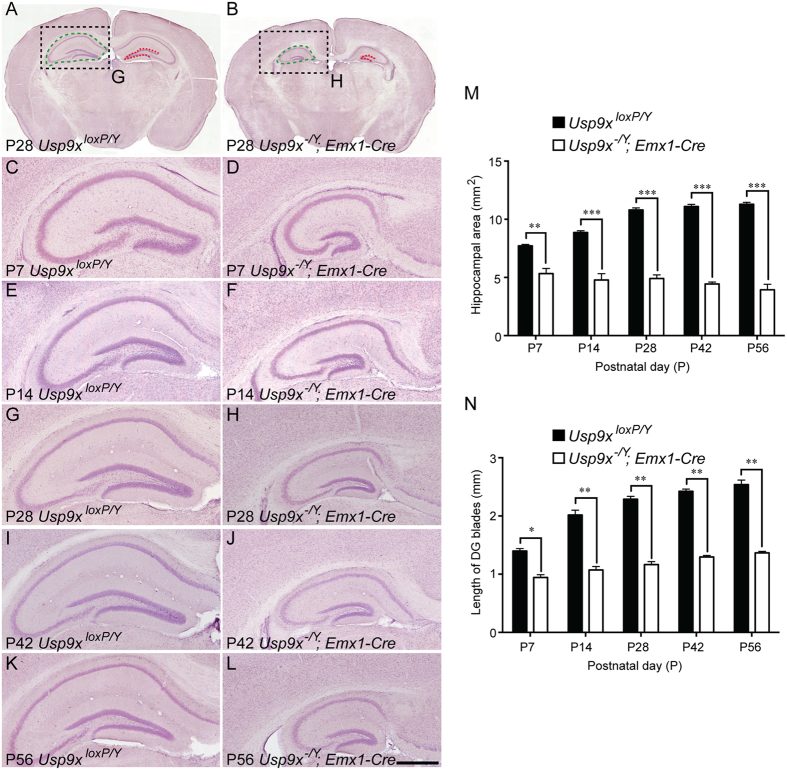
Reduced hippocampal size in postnatal and adult *Usp9x*^*−/Y*^*; Emx1-Cre* mice. Haematoxylin stained coronal sections of *Usp9x*^*loxP/Y*^ (**A,C,E,G,I,K**) and *Usp9x*^*−/Y*^*; Emx1-Cre* (**B,D,F,H,J,L**) brains at P7 (**C,D**), P14 (**E,F**), P28 (**A,B,G,H**), P42 (**I,J**) and P56 (**K,L**). (**A,B**) Low magnification views of P28 control and *Usp9x*-deficient sections. Hippocampal area was determined by measuring the extent of this structure as shown with the dotted green lines. The dotted red lines demarcate the superior and inferior blades of the dentate gyrus. (**C,D**) At P7, the mutant had a markedly smaller hippocampus, and reduced length of the blades of the dentate gyrus. The decrease in hippocampal size within *Usp9x*^*−/Y*^*; Emx1-Cre* mice was evident at each of the ages investigated (**C–L**). (**M**) Quantification of the hippocampal area revealed that the size of the mutant hippocampus was significantly reduced in comparison to the control at each of the ages studied. (**N**) Similarly, the total length of the blades of the dentate gyrus was reduced in mutant mice between P7 and P56. ^*^p < 0.05; ^**^p < 0.01; ^***^p < 0.001, *t*-test. Scale bar in (**L**): A, B −800 μm; (**C–L**) −150 μm.

**Figure 3:**
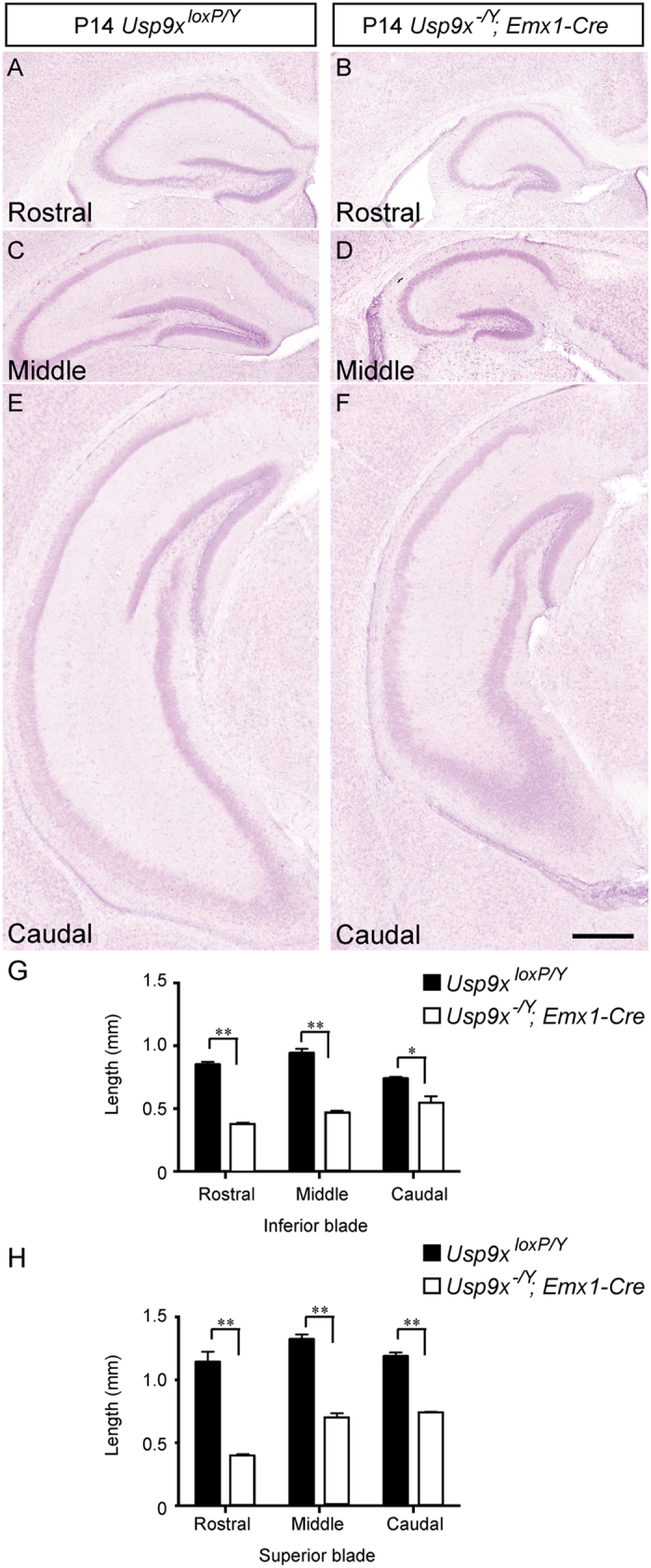
Reduced hippocampal size at different rostro-caudal levels of P14 *Usp9x*^*−/Y*^*; Emx1-Cre* mice. Haematoxylin stained coronal sections of *Usp9x*^*loxP/Y*^ (**A,C,E**) and *Usp9x*^*−/Y*^*; Emx1-Cre* (**B,D,F**) brains at rostral (**A,B**), middle (**C,D**) and caudal (**E,F**) levels of P14 hippocampi. The hippocampus of *Usp9x*^*−/Y*^*; Emx1-Cre* mice was significantly reduced at each of these levels at this age in comparison to the controls. (**G,H**) Quantification of the length of the inferior (**G**) and superior (**H**) blades of the dentate gyrus revealed that both blades were significantly reduced in length in mutant mice at rostral, middle and caudal levels in comparison to the controls. ^*^p < 0.05; ^**^p < 0.01, *t*-test. Scale bar in (**F**): 150 μm.

The subtle hippocampal phenotype of embryonic mice lacking *Usp9x*^15^, coupled with the postnatal expression of this gene (Fig. 1) and the severe reduction in hippocampal size in adult *Usp9x*^*−/Y*^*; Emx1-Cre* mice^15^ suggests that postnatal hippocampal development is aberrant in mice lacking this DUB. To test this hypothesis, we compared the hippocampal size of *Usp9x-*deficient and control mice at postnatal ages ranging from P7 to P56. Haematoxylin labeled sections revealed a significant reduction in hippocampal area in *Usp9x*^*−/Y*^*; Emx1-Cre* mice from as early as P7 in comparison to control *Usp9x*^*loxP/Y*^ mice (Fig. 2A–L, Supp. Fig. 1). Indeed, the hippocampus of *Usp9x*^*−/Y*^*; Emx1-Cre* mice was significantly smaller than controls at each of the ages analysed (Figs 2M and 3, Supp. Figs 1 and 2). Moreover, the size of the mutant hippocampus was at its greatest at P7, unlike controls, which increased in size at each age (Fig. 2M). Analysis of the combined length of the superior and inferior blades of the dentate gyrus revealed a similar trend, with pooled blade length being significantly reduced in comparison to controls (Fig. 2N), and not increasing markedly after P7. Analysis of the individual blades of the dentate gyrus also showed that both the superior and inferior blades were reduced in size at different rostro-caudal levels in the mutant in comparison to the controls postnatally and in the adult brain (Fig. 3, Supp. Figs 1 and 2). Given that the majority of the granule neurons within the dentate gyrus are produced postnatally^18^, these findings are illustrative of deficits in neurogenesis within the dentate gyrus contributing to the reduction in hippocampal size in *Usp9x*^*−/Y*^*; Emx1-Cre* mice.

**Figure 4:**
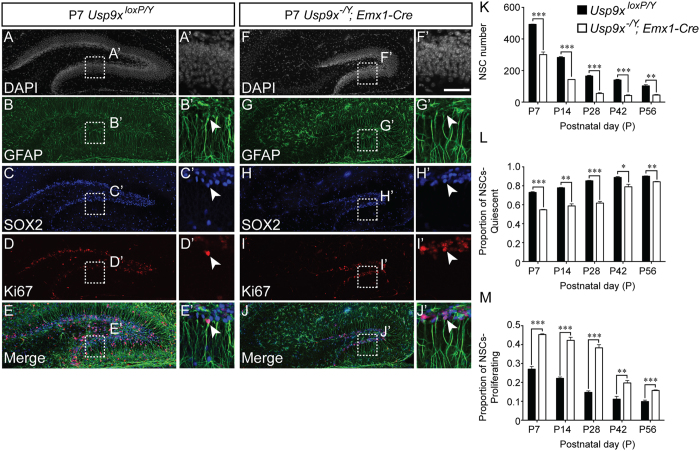
Fewer neural stem cells are present in the dentate gyrus of *Usp9x*^*−/Y*^*; Emx1-Cre* mice. (**A–J**) Co-immunofluorescence staining and confocal microscopy was performed on hippocampal sections of *Usp9x*^*loxP/Y*^ (**A–E**) and *Usp9x*^*−/Y*^*; Emx1-Cre* (**F–J**) at P7. Cell nuclei were labeled with DAPI (**A,F**). Neural stem cells (NSCs) were defined as cells expressing both GFAP ((**B,G**) green) and SOX2 ((**C,H**) blue). Proliferating NSCs were further identified by the expression of Ki67 ((**D,I**); red). The merged panels are shown in (**E,J**). The insets reveal proliferating NSCs (arrowheads) in the dentate gyrus of control and mutant animals. Quantification of NSC number revealed significantly fewer NSCs (GFAP^+^/SOX2^+^) within the dentate gyrus of *Usp9x*^*−/Y*^*; Emx1-Cre* mice at each of the ages studied (**K**). Of the NSC pool, the proportion that was quiescent (GFAP^+^/SOX2^+^/Ki67^−^) was reduced at each age in the dentate gyrus of mutant mice (**L**), whereas the proportion of NSCs that were proliferating in the mutant (GFAP^+^/SOX2^+^/Ki67^+^) was significantly increased in comparison to the controls (**M**). ^*^p < 0.05; ^**^p < 0.01; ^***^p < 0.001, *t*-test. Scale bar in F’: (**A–J**) −150 μm; A’–J’ −30 μm.

The first 3 postnatal weeks of development are critical for the development of the rodent dentate gyrus, as this is the time in which the neural stem cells coalesce into the SGZ and become quiescent^16^. Entry of stem cells into quiescence is important to ensure that this population of cells does not become prematurely depleted, so ensuring a substrate for neurogenesis throughout life^19^. Given the failure of the dentate gyrus blades to develop postnatally, we investigated neural progenitor cell number, as well as quiescence, using co-immunofluorescence labelling and confocal microscopy (a schematic of basic hippocampal structure, as well hippocampal cell types and specific markers that label these cells can be found in panels C–E respectively of Fig. 1). Progenitor cells were identified as those cells expressing both GFAP and SOX2, and proliferation was assessed using the marker Ki67. Although the total NSC number per unit area was not significantly different (data not shown), in total there were significantly fewer neural progenitor cells (GFAP^+^/SOX2^+^) within the dentate gyrus of *Usp9x*^*−/Y*^*; Emx1-Cre* mice at each of the ages analysed in comparison to controls (Fig. 4). This is in line with the dramatic reduction in dentate gyrus size in mutant mice (Fig. 2). More interestingly, when we partitioned neural progenitor cells into quiescent (GFAP^+^/SOX2^+^/Ki67^−^) and proliferating (GFAP^+^/SOX2^+^/Ki67^+^) we saw a significantly lower proportion of quiescent cells, and a concomitantly higher proportion of proliferating cells within the dentate gyrus of *Usp9x*^*−/Y*^*; Emx1-Cre* mice at each of the ages analysed (Fig. 4). From this we infer that there is a premature depletion of neural progenitor cells from the dentate gyrus of postnatal *Usp9x*^*−/Y*^*; Emx1-Cre* mice, consistent with previous reports identifying *Usp9x* as a factor promoting ‘stemness’^9^,^13^,^14^.

**Figure 5:**
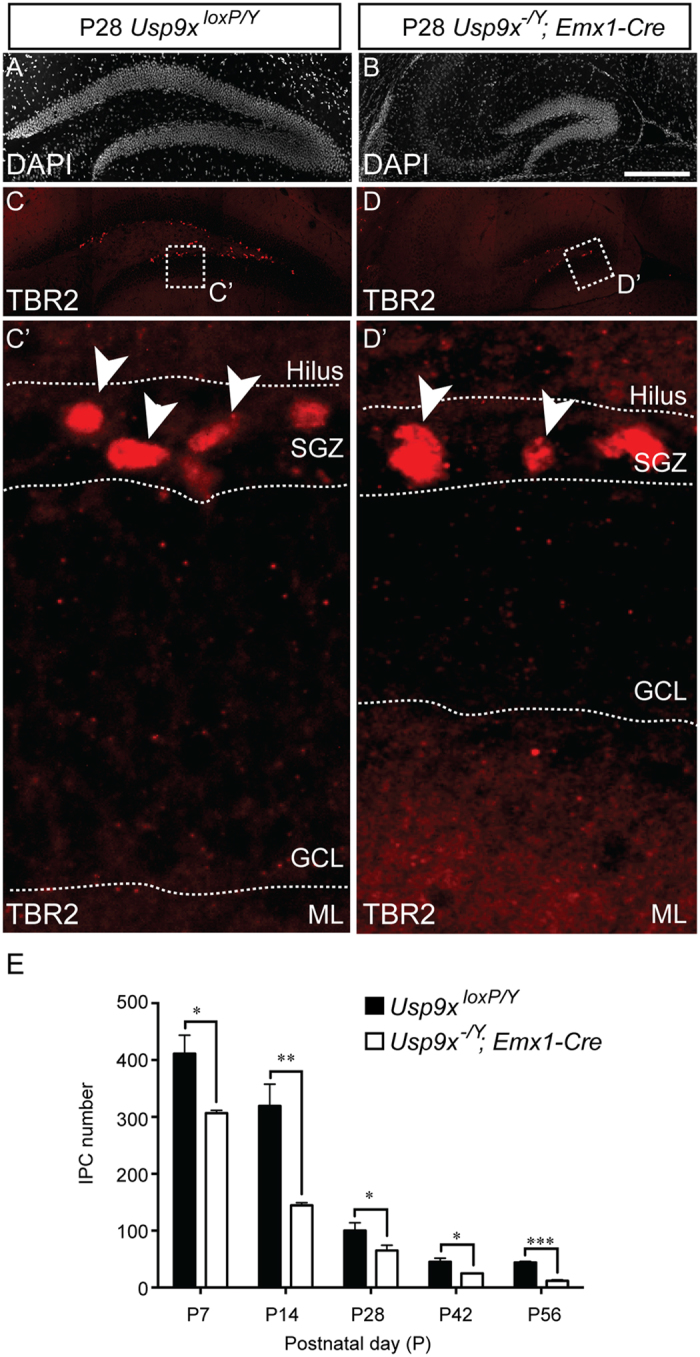
IPC number is reduced in the dentate gyrus of *Usp9x*^*−/Y*^*; Emx1-Cre* mice. (**A,B**) The dentate gyrus of P28 *Usp9x*^*loxP/Y*^ (**A**) and *Usp9x*^*−/Y*^*; Emx1-Cre* (**B**) are shown via DAPI labeling. (**C,D**) Sections were labeled with antibodies against TBR2, a marker specific for IPCs (red). The boxed regions are shown in panels C’ and D’. IPCs (arrowheads in C’,D’) were seen within the subgranular zone (SGZ). Quantification revealed that there were significantly fewer IPCs in the dentate gyrus of *Usp9x*^*−/Y*^*; Emx1-Cre* mice at each of the ages investigated. ^*^p < 0.05; ^**^p < 0.01; ^***^p < 0.001, *t*-test. GCL – granule cell layer; ML – molecular layer. Scale bar in (**B**) (**A–D**) −150 μm; C’,D’ −25 μm.

**Figure 6:**
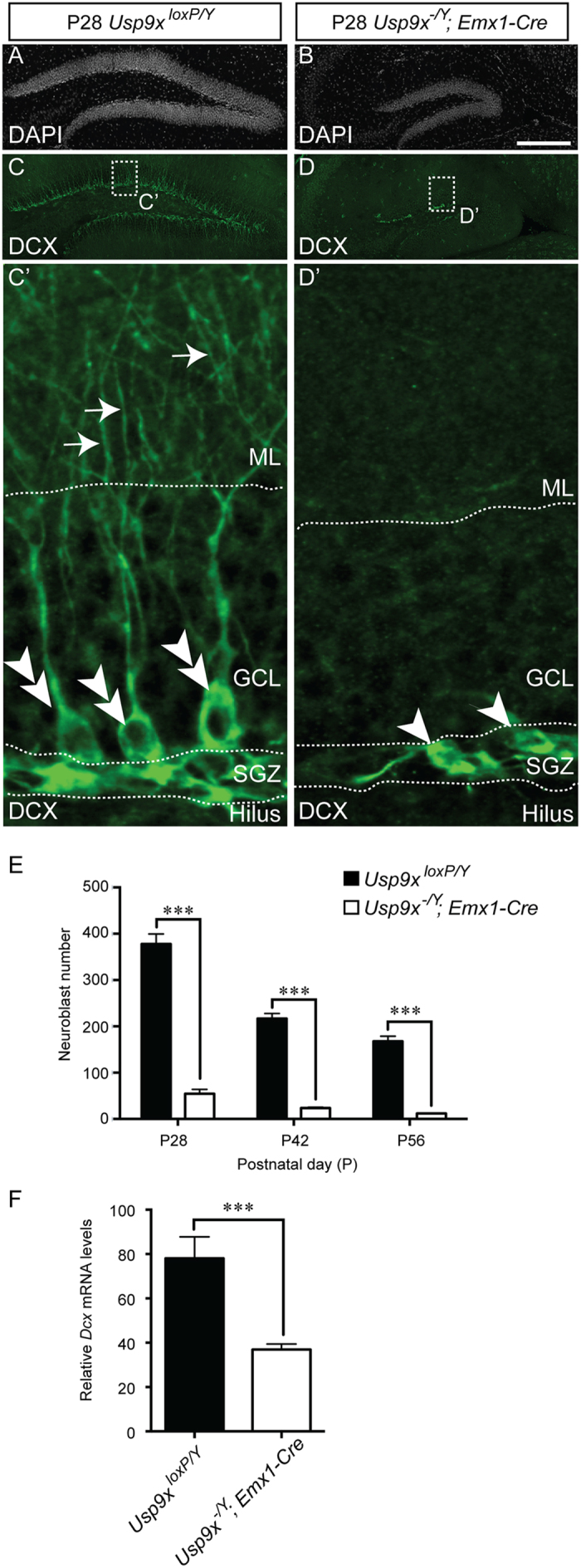
Lack of *Usp9x* culminates in deficits in neuroblast number and morphology. (**A,B**) The dentate gyrus of P28 *Usp9x*^*loxP/Y*^ (**A**) and *Usp9x*^*−/Y*^*; Emx1-Cre* (**B**) are shown via DAPI labeling. (**C,D**) Sections were labeled with antibodies against the microtubule-associated protein DCX, a marker specific for neuroblasts (green). (C’) The higher magnification view of the boxed region in (**C**) shows numerous neuroblast cell bodies above the subgranular zone (SGZ; double arrowheads in C’). These neuroblasts extend extensive processes into the molecular layer (ML, arrows in C’). (D’) In the mutant however, there were fewer of these cells (arrowheads in D’), and they did not extend processes into the ML. (**E**) There were significantly fewer neuroblasts in the dentate gyrus of *Usp9x*^*−/Y*^*; Emx1-Cre* mice in comparison to controls at P28, P42 and P56. (**F**) qPCR revealed that there were also significantly reduced levels of *Dcx* mRNA in the hippocampus of mutant mice at P14. ^***^p < 0.001, *t*-test. GCL – granule cell layer. Scale bar in (**B**) (**A–D**) −150 μm; C’,D’ −25 μm.

Proliferating neural progenitor cells within the dentate gyrus produce intermediate progenitor cells that are marked by the expression of proteins including TBR2^20^,^21^. Quantification of the number of intermediate progenitor cells within the dentate gyrus of *Usp9x*^*−/Y*^*; Emx1-Cre* and *Usp9x*^*loxP/Y*^ mice revealed that there were significantly fewer of these cells in the mutant compared to the control at each of the ages investigated (Fig. 5). Similarly, when we quantified the number of neuroblasts within the dentate gyrus (as evidenced by the expression of DCX)^22^, we observed that there were dramatically fewer neuroblasts within the mutant dentate gyrus at P28, P42 and P56 (Fig. 6A–E). Moreover, those neuroblasts that were produced in the absence of *Usp9x* exhibited an almost complete lack of dendrites, from which we infer that this DUB is critical for the extension of neuronal processes. We were unable to reliably count neuroblasts in the dentate gyrus of younger *Usp9x*^*loxP/Y*^ mice due to the density of these cells and the extent of their dendritic processes. However, qPCR analysis revealed that there was a significant reduction in *Dcx* mRNA expression in the hippocampus of mutant mice at P14 (Fig. 6F).

**Figure 7:**
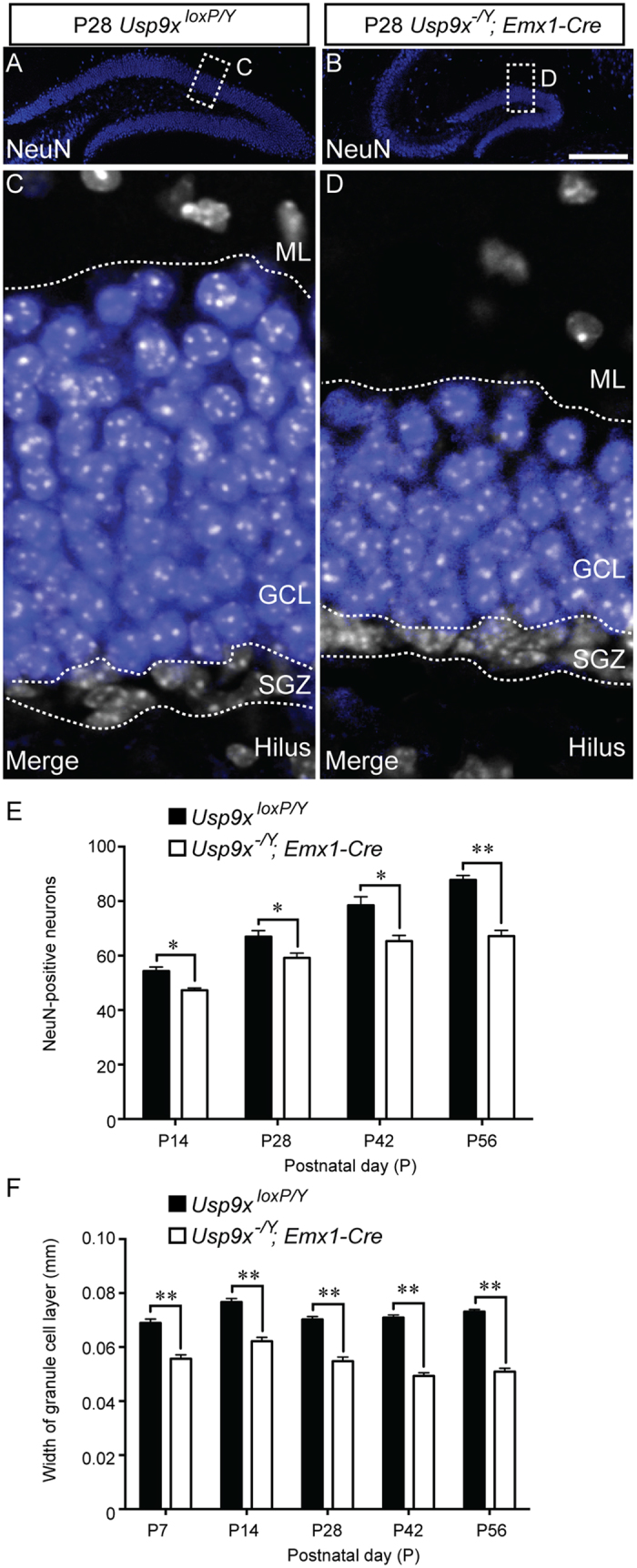
Fewer NeuN-expressing neurons in the dentate gyrus of *Usp9x*^*−/Y*^*; Emx1-Cre* mice. (**A,B**) Mature neurons within the dentate gyrus of P28 *Usp9x*^*loxP/Y*^ (**A**) and *Usp9x*^*−/Y*^*; Emx1-Cre* (**B**) are shown via NeuN labeling (blue). (**C,D**) Higher magnification views of the boxed regions in (**A,B**) respectively. These panels show the expression of NeuN (blue) and DAPI (white), revealing that the granule cell layer (GCL) is markedly thinner in mutant mice in comparison to controls. (**E**) Quantification of the number of NeuN-expressing neurons within the dentate gyrus revealed that there were significantly fewer neurons in the mutant at each of the ages studied. (**F**) Analysis of the width of the granule cell layer of the dentate gyrus revealed that this was significantly reduced in the mutant in comparison to controls at each of the ages assessed. ^*^p < 0.05; ^**^p < 0.01, *t*-test. SGZ – subgranular zone; ML – molecular layer. Scale bar in (**B**) (**A**,**B**) −150 μm; (**C**,**D**) −25 μm.

The final step in neurogenesis within the postnatal and adult dentate gyrus is the migration of immature neurons into the overlying granule cell layer, and their maturation, a process which entails elaboration of dendritic processes and axonal pathfinding, as well as the expression of mature neuronal markers such as NeuN^20^. Given the reduction in the length of the blades of the dentate gyrus, we posited that there would be fewer NeuN-positive neurons within the granule cell layer of *Usp9x*^*−/Y*^*; Emx1-Cre* mice in comparison to controls. At P7 we were unable to count the number of NeuN-expressing cells within the dentate gyrus of *Usp9x*^*loxP/Y*^ mice, as the density of these cells was too high to preform reliable counts. However, at P14 and beyond we saw that there was a significant reduction in the number of mature neurons within the knockout dentate gyrus compared to the control (Fig. 7A–E). Furthermore, the width of the NeuN-positive granule cell layer was also significantly reduced (Fig. 7F), although we did not observe any significant differences in the density of NeuN-positive nuclei between mutant and control samples at any of the ages analysed (data not shown). Collectively, these findings, when considered in light of the diminished numbers of neural stem cells and intermediate progenitors, are consistent with deficits in neurogenesis within the postnatal dentate gyrus being responsible, at least in part, for the reduction in the size of this critical brain region in mice lacking *Usp9x*.

## Discussion

The hippocampus is one of the few regions of the adult mammalian cerebral cortex in which neurogenesis persists throughout life^19^. This phenomenon is not confined to rodents, but has also been revealed, through the post mortem analysis of C^14^ ratios, to occur in humans^23^. Neurogenesis is central to ongoing learning and memory, and abnormalities in this process have been linked to epilepsy and cognitive decline^24^,^25^. Recent work has begun to elucidate the mechanisms regulating the development and the maintenance of neural stem cells within the dentate gyrus, and has revealed that factors intrinsic to neural stem cells are pivotal for their perdurance, as is the local niche microenvironment in which they reside^26^. However, far less is known of how the post-translational modification of proteins may influence adult neurogenesis. Here, we reveal that the DUB USP9X regulates multiple aspects of neurogenesis within the postnatal dentate gyrus, including regulating neural stem cell quiescence, as well as the production and morphological development of neuroblasts.

USP9X is expressed within the developing cerebral cortex^9^ and within the adult dentate gyrus^17^. Whereas the loss of this gene results in subtle changes to neocortical size, it culminates in a markedly smaller hippocampus within adult mice^15^ and from as early at P7 (this study). Indeed, the fact that deficits in dentate gyrus morphology are evident from as early as P7 indicates that a more rigorous analysis of the late embryonic phenotype of these mice is warranted. For instance, given that the infrapyramidal blade of the dentate gyrus develops after the suprapyramidal blade, a comprehensive analysis of blade development in younger mice may provide insights into the developmental timeline for the effects of *Usp9x*-deficiency. Conversely, over-expression of USP9X within cultured embryonic stem cell-derived neural progenitors promotes increased self-renewal capacity^9^. Collectively, these findings suggest that USP9X may regulate neural stem cell biology within the postnatal hippocampus. One way in which this could be mediated is via the regulation of cellular polarity. Hippocampal neural stem cells are highly polarised, with their cell bodies within the SGZ, and their processes extending into the granule cell layer^26^. Ablation of genes modulating polarity, such as *Apc*, results in premature exhaustion of the neurogenic niche within the dentate gyrus^27^, highlighting the importance of stem cell polarity for stem cell maintenance. Interestingly, USP9X promotes the polarisation of neural progenitor cells *in vitro*^9^, and co-localises with highly polarised proteins including β-catenin, p120 catenin, ZO-1 and E-cadherin in cultured intestinal cells^28^,^29^,^30^. The Par family of proteins is also central to apico-basal cellular polarity^31^, and the Par-1 homolog MARK4 is a substrate for USP9X activity^32^. Together, these findings support the notion that USP9X may play a role in the induction and/or maintenance of stem cell polarity with the nascent SGZ.

Neural stem cell quiescence is also important to ensure that the stem cell niche does not become prematurely depleted within the adult brain^33^. Recent research has shown that signals emanating from the vasculature^34^ as well as from the extracellular matrix^35^ modulate cellular quiescence in adult neural stem cell niches, as does neuronal activity^36^,^37^. At a molecular level, numerous factors have also been shown to mediate neural stem cell quiescence, including members of the Notch signalling pathway^38^,^39^, the cyclin-dependent kinase inhibitor p27^kip1^ 40,41,42, the chromatin remodelling factor CHD7^43^ and the transcription factor NFIX^44^. Could USP9X also contribute to cellular quiescence *in vivo*? Evidence suggests that this is possible, as this DUB can potentially modulate Notch pathway activity through its interactions with elements of this pathway, including Epsin^45^, Itch^7^ and mind bomb 1^46^. Moreover, UPS9X has been shown to interact with SOX2, a transcription factor localised to quiescent adult neural stem cells^20^,^47^. Future work aimed at deciphering how USP9X may mediate both polarity and quiescence in neural stem cells within the adult dentate gyrus will enable greater mechanistic insights into the *in vivo* role of this DUB. This work will be assisted through the use of Cre-drivers that will enable the ablation of *Usp9x* specifically from adult neural progenitor cells, such as a *nestin-Cre*^*ERT2*^ allele^48^, coupled with proteomic approaches aimed at determining USP9X substrates and interactors within stem cell populations. The use of different Cre-drivers, such as *CaMKIIα-Cre*, which drives recombination within neurons of the forebrain, including the CA regions of the hippocampus^49^, will further elucidate the role of USP9X within the broader hippocampal formation. Indeed, *Usp9x* mRNA is very highly expressed within the stratum pyramidale of the postnatal hippocampus (Fig. 1), and the loss of *Usp9x* culminates in the entire hippocampus being reduced in size (Fig. 2). These data are suggestive of a role for USP9X in modulating the activity of post-mitotic neurons within the CA regions of the hippocampus, but whether this pertains to the development, maturation and/or survival of these neurons remains unresolved.

Our findings also illustrate that the morphology of neuroblasts within the postnatal hippocampus is severely abnormal in the absence of *Usp9x*. Why this occurs is unclear, but previous research has provided potential insights^6^. For instance, the elaboration of neuronal processes from cultured hippocampal neurons lacking *Usp9x in vitro* is reduced, and neocortical neuronal projections are abnormal in *Usp9x*^*−/Y*^*; Emx1-Cre* mice. This is suggestive of a role for USP9X in mediating cytoskeletal dynamics and process outgrowth, a concept supported by findings revealing that USP9X interacts with DCX within the brain. The molecular logic underpinning this interaction, and how loss of USP9X disrupts cytoskeletal dynamics are at this point unknown, but work aimed at detailing the significance of the USP9X/DCX interaction will be critical to clarify this facet of USP9X function.

The implications of these data may also extend to humans, where mutations in *USP9X* are associated with neurodevelopmental disorders. Loss of USP9X function has been implicated in intellectual disability, autism^50^, epilepsy^51^ and lissencephaly^52^. *USP9X* mutations associated with intellectual disability were unable to restore axonal growth and neuroblast migration when expressed in *Usp9x*-null hippocampal neurons derived from knock-out mice, whereas wild-type *USP9X* could^50^. Therefore, interrogating the molecular basis whereby USP9X regulates postnatal hippocampal development may provide insight into the normal development of both mouse and human brain.

## Methods

### Ethics statement

All mouse breeding was performed under the ethical clearance approved by Griffith University Animal Ethics Committee (ESK 06 14 and BPS 02/11). The work performed in this study also conformed to The University of Queensland’s Animal Welfare Unit guidelines for animal use in research (AEC approval number: QBI/353/13/NHMRC). The study followed the Australian Code of Practice for the Care and Use of Animals for Scientific Purposes.

### Animal breeding

*Usp9x*^*loxP/loxP*^ female mice were crossed with *Emx1-Cre* males as described previously^15^. As *Usp9x* is located on the X chromosome, the male offspring from this cross that inherited the *Emx1-Cre* allele lacked *Usp9x* (hereafter referred to as *Usp9x*^*−/Y*^*; Emx1-Cre*). Cre-negative males were used as controls (hereafter referred to as *Usp9x*^*loxP/Y*^). Female mice were not used in this study. Genotyping was performed via PCR^15^. The day of birth was designated as postnatal (P) day 0. Mice were collected at P7, P14, P28, P42 and P56 (n ≥ 3 mice per genotype per age). Interestingly, adult *Usp9x*^*−/Y*^*; Emx1-Cre* mice were slightly, but significantly, lighter than their control counterparts (*Usp9x*^*loxP/Y*^ −25.67 ± 0.58 g; *Usp9x*^*−/Y*^*; Emx1-Cre* −23.43 ± 0.54 g; *p* < 0.05, *t-*test), though the reason underlying this is unclear. All experiments were performed in accordance with the Australian Code of Practice for the Care and Use of Animals for Scientific Purposes, and were carried out in accordance with The University of Queensland Institutional Biosafety committee.

### Immunofluorescence labelling

Mice were perfused transcardially with phosphate-buffered saline (PBS), followed by 4% paraformaldehyde (PFA), then post-fixed for 48–72 hours before long term storage in PBS at 4 °C as described previously^53^. Brains were embedded in noble agar and sectioned in a coronal plane at 50 μm using a vibratome. Sections were placed sequentially across the wells of a 6-well plate to ensure appropriate sampling from different rostro-caudal regions of the hippocampus. For the smallest brains (P7), there were 5 sections containing the hippocampus in each well. For the larger brains there were between 6–10 hippocampal sections per well. Thus, for all analyses we had at least 5 sections per animal to image and count, with each section also containing the left and right hippocampi. Sections were mounted on slides before heat-mediated antigen retrieval was performed in a 10 mM sodium-citrate solution (pH 6.0) at 95 °C for 15 minutes as described^54^. Sections were incubated for 2 hours in a blocking solution [2% vol/vol normal goat or donkey serum (Vector Laboratories) with 0.2% vol/vol Triton-X 100 in PBS] containing serum that matched the species in which the secondary antibody was raised. Primary antibodies diluted in blocking solution were applied to sections and incubated overnight at 4 °C. Following overnight incubation, sections were washed in PBS before the corresponding secondary antibodies were diluted in blocking solution and applied to sections for 2 hours. Sections were counter- stained with 4′,6-diamidino-2-phenylindole (DAPI) for 10 minutes and coverslipped using fluorescent mounting medium (DAKO).

### Antibodies

The following primary antibodies, and the dilutions at which they were used in this study were: anti-GFAP (rabbit polyclonal, 1:1000, DAKO, #Z0334), anti-Ki67 (mouse monoclonal, 1:200, BD Pharmingen Biosciences, #556003), conjugated anti-SOX2 Alexa Fluor 488 (rat monoclonal, 1:400, eBioscience, #53-9811), anti-DCX (goat polyclonal, 1:200, Santa Cruz Biotechnology, #sc-8066), anti-TBR2 (rabbit polyclonal, 1:200, Abcam, #ab23345), anti-NeuN (mouse monoclonal, 1:400, Millipore, #MAB377).

### Haematoxylin staining

Haematoxylin staining was performed using Mayer’s Haematoxylin solution (Sigma-Aldrich) using standard protocols^55^.

### *In situ* hybridisation

A *Usp9x* riboprobe was created by amplifying a fragment of the *Usp9x* gene using the following primers: Forward: 5′AGGAAGCGGTTCTCAGTTACAC3′, Reverse: 5′AGACAGGCAGACAGAGAAGGT3′. The fragment was cloned into the vector pGEM-T Easy (Promega), and anti-sense and sense riboprobes were generated using a DIG RNA labeling kit (Roche). *In situ* hybridisation was conducted as described previously^56^ with minor modifications. Sections were subjected to heat-mediated antigen retrieval, and hybridisation was conducted at 70 °C. The colour reaction was performed with BM Purple (Roche).

### Image acquisition and analysis

All brightfield images were captured using Axio Imager Z2 upright microscopes (Zeiss) attached to a digital camera (Zeiss AxioCam HRc). All fluorescent images were captured on an inverted spinning-disk confocal system using a 20 X objective (Axio Observer Z1 Carl Zeiss; CSU-W1 Yokogawa Corporation of America). We took 10 consecutive 1 μm-thick optical sections to generate a 10 μm-thick z-stack. In all cases the 10 μm z-stack was taken from the middle of the section to minimize potential artefacts arising from the sectioning process such as damage to the tissue. Image acquisition was performed using SlideBook 6.0 (3I, Inc). Hippocampal area measurements and cell quantification were completed using Image J. To measure hippocampal area and the lengths of the dentate gyrus blades, images were taken of haematoxylin-stained sections, and quantification of area and length were performed in ImageJ. Measurements were performed on the left and right dentate gyri from both rostral, middle and caudal regions of the hippocampus. Hippocampal area, as well as the length of the dentate gyrus blades, was reduced within rostral, middle and caudal hippocampal sections of mutant mice in comparison to controls (Fig. 3, Supp. Figs 1 and 2). As such, data from different rostro-caudal sections of *Usp9x*^*loxP/Y*^ and *Usp9x*^*−/Y*^*; Emx1-Cre* mice were pooled to provide the mean values presented in Fig. 2.

For cell counts, the following cellular populations were analysed: quiescent NSCs (GFAP^+^/SOX2^+^/Ki67^−^), proliferating NSCs (GFAP^+^/SOX2^+^/Ki67^+^), IPCs (TBR2^+^) and neuroblasts (DCX^+^). Cell counts were performed in both the left and right dentate gyrus for each section. To perform the cell counts, images were viewed in Image J. Immuno-positive cells in the first optical section were counted, and were marked using the counting tool in this program. The next optical section was then overlaid over the first, such that cells that had already been counted were still visible and marked with the counting tool. Counts were then performed on the second optical section. We then proceeded to do this with the remaining sections within the z-stack. In this way we were able to avoid counting cells twice. Cell counts per unit area of the dentate gyrus were also calculated (data not shown), but given the markedly reduced size of the mutant dentate gyrus, we chose to present the data as cell numbers within the z-stack per dentate gyrus as this was more reflective of the phenotype observed. We did not see any significant changes in the cell counts performed at different rostro-caudal levels in either control or knockout sections (data not shown). Mature neurons (NeuN^+^) were quantified in a similar fashion, with the exception that the entire dentate gyrus was not analysed, due to the large number of immuno-positive cells. Instead, four sampling areas, each of which was 50 μm wide running parallel to the SGZ, were used (two in the upper blade, and two in the lower blade, of the dentate gyrus). Supplementary Fig. 3 provides a schematic depicting the sampling areas selected. Counts were performed on dentate gyri from rostral, middle and caudal regions of the hippocampus, and were pooled to provide the mean values presented. The density of NeuN-positive nuclei was not significantly different between mutant and control cohorts at the ages analysed (data not shown). Samples were stained and imaged simultaneously, and cell counts were performed blind to the genotype of the sample.

### Quantitative real-time PCR

Quantitative real-time PCR (qPCR) was performed using standard protocols as described previously^57^. Briefly, the hippocampal formation was microdissected from the hippocampus of P14 *Usp9x*^*−/Y*^*; Emx1-Cre* and *Usp9x*^*loxP/Y*^mice^58^ and snap frozen. An RNeasy Micro Kit (Qiagen) was used to isolate total RNA. Superscript III (Invitrogen) was used to perform reverse transcription on total RNA (0.5 μg) using random hexamers. qPCR reactions were carried out in a Rotor-Gene 3000 (Corbett Life Science) using the SYBR Green PCR Master Mix (Invitrogen). All of the samples were diluted to 1:100 with RNase/DNase-free water and 5 μL of these dilutions were used for each SYBR Green qPCR reaction that also contained 10 μL SYBR Green PCR Master Mix, 10 μM of each primer and deionised water. The reactions were incubated for 10 minutes at 95 °C followed by 40 cycles with 15 seconds denaturation at 95 °C, 20 seconds annealing at 60 °C and 30 seconds extension at 72 °C. For this analysis, we isolated the hippocampi from 4 *Usp9x*^*loxP/Y*^ mice, and 6 *Usp9x*^*−/Y*^*; Emx1-Cre* mice. All the samples were tested in triplicate, and each experiment was repeated a minimum of three times. The house keeping gene *glyceraldehyde 3-phosphate dehydrogenase* was used to calculate relative changes in gene expression level, which is presented as ^−^ΔΔCt. The primer sequences used were: *Dcx* For: 5′TGGAAGCATGGATGAACTGG3′; *Dcx* Rev: 5′CATGTTGGCAGATGTCTTTACG3′; *Gapdh* For: 5′GCACAGTCAAGGCCGAGAAT3′; *Gapdh* Rev: 5′GCCTTCTCCATGGTGGTGAA3′.

### Statistical analysis

Student’s *t*-tests were used to compare the datasets described here. For all haematoxylin and immunocytochemical analyses, we used n = 3 animals per age, per genotype. For the qPCR analysis, we interrogated mRNA from 4 biological replicates for the *Usp9x*^*loxP/Y*^group, and 6 biological replicates for the *Usp9x*^*−/Y*^*; Emx1-Cre* group. Statistical significance was established at a *p*-value of <0.05. Error bars represent standard error of the mean (SEM). Data analysis was performed blind to the genotype of the sample. The mean and SEM of all the data points reported in this study are provided in Supplementary Table 1.

## Additional Information

**How to cite this article**: Oishi, S. *et al*. *Usp9x*-deficiency disrupts the morphological development of the postnatal hippocampal dentate gyrus. *Sci. Rep.* **6**, 25783; doi: 10.1038/srep25783 (2016).

## Supplementary Material

Supplementary Information
