# Supplementary material for: Usp9x-deficiency disrupts the morphological development of the postnatal hippocampal dentate gyrus
Source: Sci Rep. 2016 May 16;6:25783. doi: 10.1038/srep25783 (PMC4867638; doi:10.1038/srep25783)
Supplement: Supplementary Information [file srep25783-s1.pdf]

Supplementary Information

Usp9x-deficiency disrupts the morphological development of the postnatal hippocampal dentate gyrus

Sabrina Oishi[1], Susitha Premarathne[2], Tracey J. Harvey[1], Swati Iyer[1], Chantelle Dixon[1], Suzanne Alexander[3,4], Thomas H. J. Burne[3,4], Stephen A. Wood[2, Ψ], Michael Piper[1,4, Ψ].

[1]The School of Biomedical Sciences and [4]Queensland Brain Institute, The University of Queensland, Brisbane, QLD, Australia, 4072.
[2]The Eskitis Institute for Drug Discovery, Griffith University, Brisbane, QLD, Australia, 4111.
[3]Queensland Centre for Mental Health Research, The Park Centre for Mental Health, Richlands, QLD, Australia, 4077.

[Ψ] Authors for correspondence

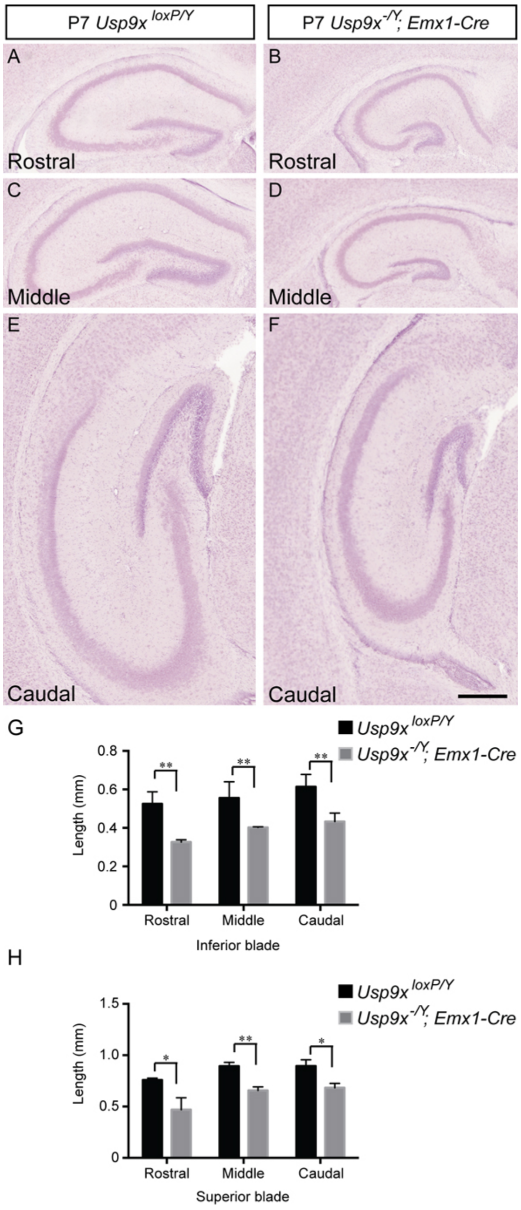

Supplementary figure 1. Reduced hippocampal size at different rostro-caudal levels of P7 *Usp9x*$^{-/Y}$*; Emx1-Cre* mice.

Haematoxylin stained coronal sections of *Usp9x*$^{loxP/Y}$ (***A, C, E,***) and *Usp9x*$^{-/Y}$*; Emx1-Cre* (***B, D, F***) brains at rostral (***A, B***), middle (***C, D***) and caudal (***E, F***) levels of P7 hippocampi. The hippocampus of *Usp9x*$^{-/Y}$*; Emx1-Cre* mice was significantly reduced at each of these levels at this age in comparison to the controls. (***G, H***) Quantification of the length of the inferior (***G***) and superior (***H***) blades of the dentate gyrus revealed that both blades were significantly reduced in length in mutant mice at rostral, middle and caudal levels in comparison to the controls. * $p < 0.05$; ** $p < 0.01$, *t*-test. Scale bar in ***F***: 125 μm.

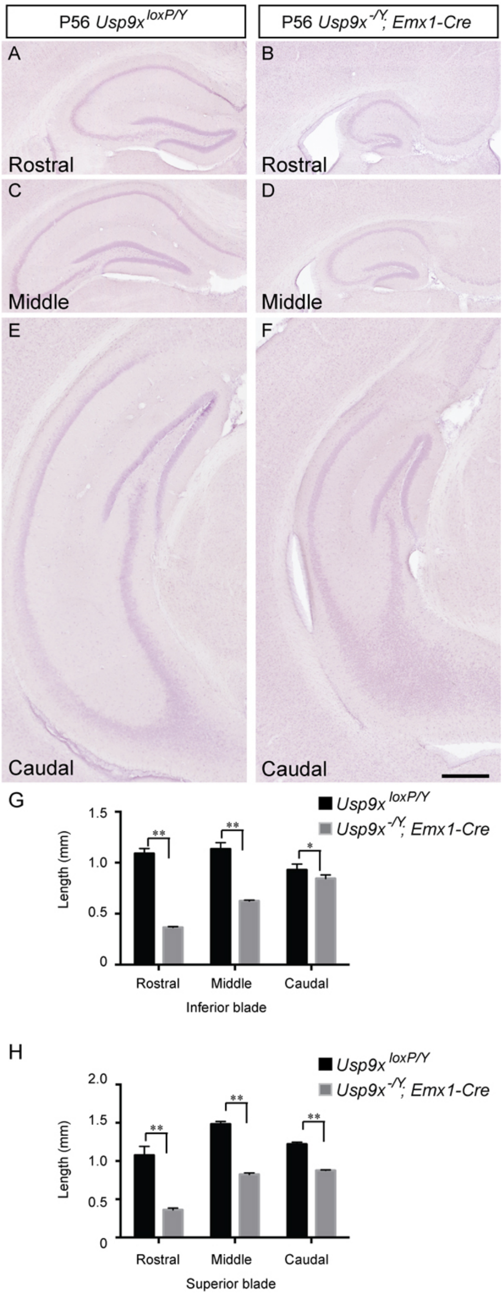

Supplementary figure 2. Reduced hippocampal size at different rostro-caudal levels of P56 *Usp9x*$^{-/Y}$*; Emx1-Cre* mice.

Haematoxylin stained coronal sections of *Usp9x*$^{loxP/Y}$ (***A, C, E,***) and *Usp9x*$^{-/Y}$*; Emx1-Cre* (***B, D, F***) brains at rostral (***A, B***), middle (***C, D***) and caudal (***E, F***) levels of P56 hippocampi. The hippocampus of *Usp9x*$^{-/Y}$*; Emx1-Cre* mice was significantly reduced at each of these levels at this age in comparison to the controls. (***G, H***) Quantification of the length of the inferior (***G***) and superior (***H***) blades of the dentate gyrus revealed that both blades were significantly reduced in length in mutant mice at rostral, middle and caudal levels in comparison to the controls. * $p < 0.05$; ** $p < 0.01$, *t*-test. Scale bar in ***F***: 150 µm.

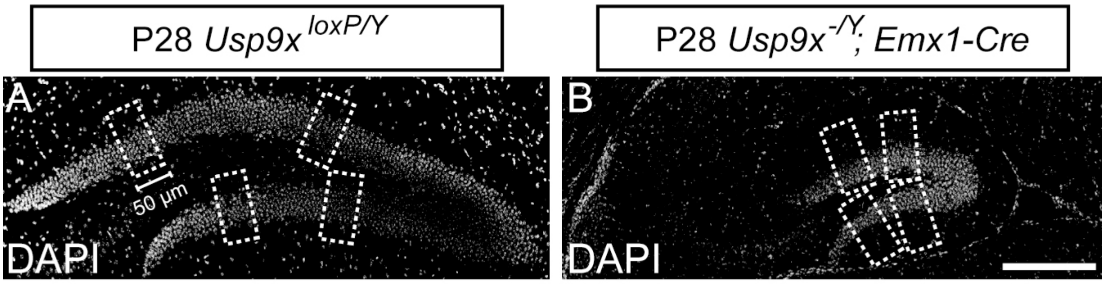

Supplementary figure 3. Sampling areas selected for NeuN cell counts.

DAPI-stained coronal sections of *Usp9x*$^{loxP/Y}$ (***A***) and *Usp9x*$^{-/Y}$*; Emx1-Cre* (***B***) brains at P28. Four sampling areas were used to perform the counts of NeuN-positive cells, two in the superior blade of the dentate gyrus, and two in the inferior blade (see boxes in ***A***, ***B***). The sampling areas were 50 µm in width. The position of these sampling areas was applied consistently on sections from rostral, middle and caudal regions from the hippocampus, and across the ages analysed in this study. Scale bar in ***B***: 150 µm.

Supplementary Table 1

Experiment			Figure	Mean	SEM ±	*p* value	*t* score	*n* number	degrees of freedom	Sections per animal	Test
Measurement/Cell type	Age	Genotype									
Area of hippocampus	P7	WT	2M	7.71578	0.1197308	0.001071	8.45737	3	4	5	t-test
	P7	cKO		5.3312	0.2552689			3		5	
	P14	WT		8.86509	0.1571322	0.0003069	11.6828	3	4	6	t-test
	P14	cKO		4.78112	0.3122658			3		6	
	P28	WT		10.8091	0.1717684	1.806E-05	23.9393	3	4	8	t-test
	P28	cKO		4.90908	0.1767398			3		8	
	P42	WT		11.0991	0.1849973	5.877E-06	31.7351	3	4	10	t-test
	P42	cKO		4.43857	0.0991248			3		10	
	P56	WT		11.288	0.1679516	2.203E-05	22.7705	3	4	10	t-test
	P56	cKO		3.93961	0.2755688			3		10	
Length of dentate gyrus blades	P7	WT	2N	1.39734	0.0436201	0.0144748	8.22126	3	4	5	t-test
	P7	cKO		0.943044	0.0339237			3		5	
	P14	WT		2.01612	0.0839241	0.0098623	9.99495	3	4	6	t-test
	P14	cKO		1.07171	0.043415			3		6	
	P28	WT		2.28806	0.0493635	0.0029037	18.5172	3	4	8	t-test
	P28	cKO		1.16661	0.035087			3		8	
	P42	WT		2.42489	0.0371095	0.0013305	27.3885	3	4	10	t-test
	P42	cKO		1.29533	0.017995			3		10	
	P56	WT		2.53898	0.077216	0.0045231	14.8186	3	4	10	t-test
	P56	cKO		1.36573	0.0175025			3		10	
Neural stem cell number	P7	WT	3K	492.4	2.294195	0.0003444	11.3434	3	4	5	t-test
	P7	cKO		300.633	16.74916			3		5	
	P14	WT		282.733	5.47306	1.5E-05	25.0828	3	4	6	t-test
	P14	cKO		142.833	1.07445			3		6	
	P28	WT		164.9	4.445593	0.0001142	15.0293	3	4	8	t-test
	P28	cKO		54.4	5.856051			3		8	
	P42	WT		139.933	4.557898	8.898E-05	16.0109	3	4	10	t-test
	P42	cKO		41.8667	4.091591			3		10	
	P56	WT		102.767	7.717152	0.0024342	6.80669	3	4	10	t-test
	P56	cKO		44.8	3.601389			3		10	
Proportion of queiscent neural stem cells	P7	WT	3L	72.9861	0.8118447	3.602E-05	20.1191	3	4	5	t-test
	P7	cKO		54.6767	0.4112196			3		5	
	P14	WT		77.8586	0.4208587	0.0010238	8.55766	3	4	6	t-test
	P14	cKO		58.7235	2.196064			3		6	
	P28	WT		85.2737	0.4496884	0.0001779	13.4292	3	4	8	t-test
	P28	cKO		61.7157	1.695625			3		8	
	P42	WT		88.8483	0.8631446	0.0229293	3.59159	3	4	10	t-test
	P42	cKO		78.9526	2.61654			3		10	
	P56	WT		90.1154	0.4234312	0.0004153	10.811	3	4	10	t-test
	P56	cKO		84.2707	0.3361273			3		10	
Proportion of proliferating neural stem cells	P7	WT	3M	27.0139	0.8118448	3.602E-05	20.1191	3	4	5	t-test
	P7	cKO		45.3233	0.4112196			3		5	
	P14	WT		22.1414	0.4208609	0.0002566	12.23	3	4	6	t-test
	P14	cKO		42.2765	1.591671			3		6	
	P28	WT		14.7263	0.4496856	0.0001779	13.4292	3	4	8	t-test
	P28	cKO		38.2843	1.695625			3		8	
	P42	WT		11.1517	0.8631449	0.0052279	5.52902	3	4	10	t-test
	P42	cKO		19.714	1.285767			3		10	
	P56	WT		9.8846	0.4234274	0.0004152	10.8111	3	4	10	t-test
	P56	cKO		15.7293	0.3361285			3		10	
Intermediate progenitor cells	P7	WT	4E	411.433	18.74249	0.0056827	5.40251	3	4	5	t-test
	P7	cKO		306.667	4.977728			3		5	
	P14	WT		319.267	22.20423	0.0015197	7.71463	3	4	6	t-test
	P14	cKO		144.667	4.381146			3		6	
	P28	WT		100.144	7.968778	0.0445364	2.89059	3	4	8	t-test
	P28	cKO		65.1333	9.121465			3		8	
	P42	WT		45.4976	3.59828	0.0050245	5.59003	3	4	10	t-test
	P42	cKO		24.9074	0.7871997			3		10	
	P56	WT		44.1	1.201388	0.0001266	14.6415	3	4	10	t-test
	P56	cKO		11.8	1.850225			3		10	
Neuroblast cell count	P28	WT	5E	377.778	21.61885	0.0001642	13.705	3	4	8	t-test
	P28	cKO		54.4667	9.441988			3		8	
	P42	WT		216.708	11.22969	7.128E-05	16.9351	3	4	10	t-test
	P42	cKO		23.5352	2.001467			3		10	
	P56	WT		167.833	10.78646	0.0001347	14.4132	3	4	10	t-test
	P56	cKO		12.0333	0.7055339			3		10	
Neuroblast qPCR	P14	WT	5F	78.0939	9.693335	3.14E-05	8.37E+00	4	8	N/A	t-test
	P14	cKO		36.9321	6.036714			6		N/A	
Mature Neurons	P14	WT	6E	54.338	1.508542	0.0149032	4.09588	3	4	6	t-test
	P14	cKO		47.2722	0.8367876			3		6	
	P28	WT		66.9833	2.177697	0.0485358	2.80556	3	4	8	t-test
	P28	cKO		59.1583	1.742624			3		8	
	P56	WT		87.775	1.655736	0.0016044	7.60484	3	4	10	t-test
	P56	cKO		67.15	2.148012			3		10	
Width of granule cell layer	P7	WT	6F	0.0689366	0.0014618	0.002978	6.4766	3	4	5	t-test
	P7	cKO		0.0556932	0.0556932			3		5	
	P14	WT		0.0763526	0.0015982	0.0028219	6.54187	3	4	6	t-test
	P14	cKO		0.0621892	0.0621892			3		6	
	P28	WT		0.0702253	0.0010264	0.0011093	8.37984	3	4	8	t-test
	P28	cKO		0.0547915	0.0547915			3		8	
	P42	WT		0.0709128	0.000978	0.0001373	14.3429	3	4	10	t-test
	P42	cKO		0.0493278	0.0493278			3		10	
	P56	WT		0.0731142	0.0008106	0.0001247	14.6981	3	4	10	t-test
	P56	cKO		0.0509199	0.0509199			3		10	
